# Changes in Serum Creatinine Levels Can Help Distinguish Hypovolemic from Euvolemic Hyponatremia

**DOI:** 10.3390/medicina58070851

**Published:** 2022-06-25

**Authors:** Jorge Gabriel Ruiz-Sánchez, Martín Cuesta, Emilia Gómez-Hoyos, Jersy Cárdenas-Salas, Miguel Ángel Rubio-Herrera, Estefanía Martínez-González, Paz De Miguel Novoa, Jara Eloisa Ternero-Vega, Alfonso Luis Calle-Pascual, Isabelle Runkle

**Affiliations:** 1 Departamento de Endocrinología, Hospital Universitario Fundación Jiménez Díaz, 28040 Madrid, Spain; jersy_cardenas@hotmail.com; 2 Servicio de Endocrinología y Nutrición, Instituto de Investigación Sanitaria San Carlos (IdISSC), Hospital Clínico San Carlos, 28040 Madrid, Spain; cuestamartintutor@gmail.com (M.C.); marubioh@gmail.com (M.Á.R.-H.); pazdemiguelnovoa@gmail.com (P.D.M.N.); acallepascual@hotmail.com (A.L.C.-P.); irunkledelavega@gmail.com (I.R.); 3 Centro de Investigación Biomédica en Red de Diabetes y Enfermedades Metabólicas Asociadas (CIBERDEM), 28029 Madrid, Spain; 4 Servicio de Endocrinología y Nutrición, Hospital Clínico Universitario de Valladolid, 47003 Valladolid, Spain; emiliagomezhoyos@gmail.com; 5 Servicio de Análisis Clínicos, Instituto de Medicina de Laboratorio, Hospital Clínico San Carlos, Instituto de Investigación Sanitaria San Carlos (IdISSC), 28040 Madrid, Spain; estefania.martinez@salud.madrid.org; 6 Servicio de Medicina Interna, Hospital Universitario Virgen del Rocío, 41013 Sevilla, Spain; jaraeloisa@hotmail.com

**Keywords:** hyponatremia, hypovolemic hyponatremia, euvolemic hyponatremia, SIAD, serum creatinine

## Abstract

*Background and Objectives*: Differentiating between hypovolemic (HH) and euvolemic hyponatremia (EH) is crucial for correct diagnosis and therapy, but can be a challenge. We aim to ascertain whether changes in serum creatinine (SC) can be helpful in distinguishing HH from EH. *Materials and Methods*: Retrospective analysis of patients followed in a monographic hyponatremia outpatient clinic of a tertiary hospital during 1 January 2014–30 November 2019. SC changes during HH and EH from eunatremia were studied. The diagnostic accuracy of the SC change from eunatremia to hyponatremia (∆SC) was analyzed. *Results*: A total of 122 hyponatremic patients, median age 79 years (70–85), 46.7% women. In total, 70/122 patients had EH, 52/122 HH. During hyponatremia, median SC levels increased in the HH group: +0.18 mg/dL [0.09–0.39, *p* < 0.001], but decreased in the EH group: −0.07 mg/dL (−0.15–0.02, *p* < 0.001), as compared to SC in eunatremia. HH subjects presented a higher rate of a positive ∆SC than EH (90.4% vs. 25.7%, *p* < 0.001). EH subjects presented a higher rate of a negative/null ∆SC than HH (74.3% vs. 9.6%, *p* < 0.001). ROC curve analysis found an AUC of 0.908 (95%CI: 0.853 to 0.962, *p* < 0.001) for ∆SC%. A ∆SC% ≥ 10% had an OR of 29.0 (95%CI: 10.3 to 81.7, *p* < 0.001) for HH. A ∆SC% ≤ 3% had an OR of 68.3 (95%CI: 13.0 to 262.2, *p* < 0.001) for EH. *Conclusions*: The assessment of SC changes from eunatremia to hyponatremia can be useful in distinguishing between HH and EH.

## 1. Introduction

Hyponatremia is the most frequent electrolyte disturbance in both hospital and outpatient settings, and is associated with high risk of mortality and comorbidity [1,2].

The physiopathological mechanisms behind the development of hyponatremia can be classified according to volemic status. Thus, hyponatremia can be hypovolemic (HH), hypervolemic, or euvolemic (EH) [3]. Total body water and sodium are reduced in hypovolemic patients either due to gastrointestinal or renal losses, hemorrhages, or due to a deficit in salt and water intake. Hypervolemic patients, on the other hand, have elevated total body water and sodium, although part of this is abnormally distributed in a third space, as occurs in heart failure or liver cirrhosis. Hypervolemic and HH are characterized by a reduced effective circulating volume (ECV), whereas EH is characterized by an increase in ECV. The reduction in ECV stimulates the production and release of vasopressin via baroreceptors, which will act in the collector tubule of the nephron and will increase the reabsorption of free water to the bloodstream, inducing hyponatremia. An euvolemic status, on the other hand, should not induce the production of vasopressin. In fact, an increase in ECV is used to inhibit vasopressin production/release. Thus, when EH is present, hyponatremia can be vasopressin-dependent or not. The former is due to non-osmotic production of vasopressin, other than via baroreceptor. The latter is due to an excess of free water intake or administration. Therefore, volemic classification of hyponatremia leads to knowing its etiology. 

A correct evaluation of volemia is crucial for an adequate diagnosis and further treatment of hyponatremia [2,4,5,6,7,8]. However, volemic classification can be a challenge. Since hypervolemia is usually easily recognized by history and physical examination, the difficulty resides primarily in differentiating EH from HH. In fact, close to 50% of patients can be incorrectly classified [5].

Several tests have been proposed to be of use in differentiating euvolemia from hypovolemia in hyponatremia [9,10,11,12,13]. However, their results can be confusing, and overlap is frequent. Since the physiopathology of EH and HH depends on ECV, it is probable that other biochemical blood markers directly related with ECV can be of use in distinguishing between euvolemia and hypovolemia in the setting of hyponatremia. One proposed volemic marker is the evaluation of the changes in serum creatinine levels (SC) related with natremia [14,15]. A reduction in ECV, as occurs in HH, would induce an increase in SC [16], while a normal or increased ECV would not induce changes or would slightly decrease SC. The aim of this study is to ascertain whether modifications in SC can be helpful in distinguishing hypovolemic from euvolemic hyponatremia.

## 2. Materials and Methods

We retrospectively analyzed the adult patients with hyponatremia, followed in a specialized outpatient clinic of Hyponatremia of the Endocrinology and Nutrition Department of the Hospital Clínico San Carlos (HCSC), Madrid, Spain, attended from 1 January 2014 through 30 November 2019, since informatic registry is available from this date. Patients with hyponatremia are usually referred to our monographic clinic from other hospital specialties (both wards following discharge and outpatient clinics), emergency room (following discharge when a serum sodium (SNa) ≥ 128 mmol/L is reached), and from primary care practitioners when chronic hyponatremia is detected, persisting at 2 or more visits. However, there is not a strict protocol for derivation in our clinic system. In the current study, all hyponatremic patients who attended the clinic during the specified time period were selected for the study. The study was approved by the HCSC Research Ethics Committee (Cod. 20-020, 30 November 2020). Written informed consent was waived given the registry’s anonymized and retrospective nature.

### 2.1. Criteria of Inclusion

Patients with HH and those with EH secondary to the Syndrome of Inappropriate Antidiuresis (SIAD) were included. Hyponatremia was defined as a SNa ≤ 135 mmol/L, following correction for glycemia [17].

### 2.2. Criteria of Exclusion

Patients with a prior history of diabetes, advanced chronic kidney disease (glomerular filtrate rate below 30 mL/min), nephrotic syndrome, cirrhosis, or heart failure, as well as those with pseudohyponatremia were excluded. Patients with severe symptoms as well as those with acute hyponatremia were excluded from the study. Hyponatremic patients were diagnosed as hypovolemic when showing a maximum height of the internal jugular pulse (HIJP) below the sternal angle when reclined at 0–30° [15], as well as at least two of the following: thirst, orthostatic symptoms, hypotension (blood pressure ≤ 90/60 mmHg), tachycardia (heart rate ≥ 90 bpm), decreased eye tone on palpation, or spot urinary sodium (UNa) ≤ 30 mmol/L with a non-diluted urine (indicated by a urine osmolality (UOsm) greater than plasma) for extrarenal losses [5,6]. When HIJP was not measured, HH was diagnosed with the presence of at least three of the other clinical features described above. The frequencies of the parameters evaluated to define hypovolemia in our cohort are displayed in Table 1. Patients without signs of hypovolemia and an HIJP at 1–3 cm above the sternal angle were classified as euvolemic. SIAD was diagnosed according to clinical guidelines [1,2]: euvolemic, UNa > 30 mmol/L, plasma osmolality (POsm) < 275 mOsm/Kg, UOsm > 100 mOsm/Kg, absence of diuretic use, absence of hypothyroidism and hypocortisolism. At diagnosis, HH patients were treated with increased fluid and salt intake (at libitum), and EH patients with fluid restriction based on their thirst or with initial doses of 15 mg of tolvaptan. Following 48–72 h of therapy, those remaining with EH were instructed to restrict fluid intake to 1 L or to double the dose of tolvaptan, whereas HH patients were instructed to increase fluid intake to at least 1.5 L concomitantly with a salt consumption of at least 6 g per day. Baseline euvolemia or hypovolemia was confirmed when SNa increased by a minimum of 3 mmol/L or was normalized following the respective therapy. Additionally, baseline hypovolemia was confirmed when HIJP also rose to 1–3 cm above the sternal angle following treatment.

### 2.3. Data Collection

Clinical and laboratory data were collected during the hyponatremic episode, coinciding with the moment of volemic diagnosis. Parameters coinciding with eunatremia up to 3 months prior to the diagnosis of hyponatremia, when available, were also collected. When recent eunatremic biochemistry was missing, parameters corresponding with eunatremia up to 1 month after the hyponatremic episode was resolved were included.

The variables studied were: age, sex, SNa, corresponding SC, glycemia, serum potassium (SK), POsm, spot UNa, urinary potassium (UK) and UOsm, volemic status, origin of hypovolemia (gastrointestinal losses if there was a recent history of vomiting and/or diarrhea, urinary losses if patients presented clinical factors for urinary sodium loss (e.g., use of diuretics, proximal tubular disease, Addison’s Disease, or hypoaldosteronism) in addition to UNa > 30 mmol/L, hemorrhage, or indeterminate), and the change in SC from eunatremia to hyponatremia (∆SC). Isolated hypoaldosteronism was diagnosed when HH with urine sodium wasting was detected in the absence of diuretic use, bicarbonate administration, or persisting after withdrawal of any of aforementioned factors, and in absence of hypokalemia (SK ≤ 3.5 mmol/L). The presence of a trans-tubular potassium gradient < 7 during the episode supported the diagnosis of hypoaldosteronism. An adequate response of SNa to saline reposition and/or treatment with fludrocortisone and/or the discontinuation of medication known to induce hypoaldosteronism corroborated the diagnosis of isolated hypoaldosteronism once Addison’s Disease had been ruled out.

∆SC was calculated as shown in the following formula:

(1) $$∆\mathrm{SC}\%=\frac{\left(HNaSC-ENaSC\right)}{ENaSC}\times 100$$

where ∆SC% is the percentual change in SC from eunatremia to hyponatremia, *HNaSC* is the serum creatinine coinciding with hyponatremia, and *ENaSC* is the serum creatinine coinciding with eunatremia.

### 2.4. Statistical Analysis

Categorical variables are presented with frequency distribution. Quantitative variables are expressed as median and interquartile ranges (IQR), since the Kolmogorov-Smirnov test found a non-parametric tendency. Comparative analysis between quantitative variables was performed using Mann-Whitney U or Kruskal-Wallis tests, while for that of categorical variables the Chi-squared test was used. Additionally, correlation studies with Pearson’s method were executed for parametric, and Spearman’s method for non-parametric variables.

The main variables studied were ∆SC and ∆SC%. The area under the curve (AUC) of the receiver operator characteristic (ROC) curve was used to determine the optimal cut-off point for ∆SC and ∆SC% for differentiation of HH from EH. Positive predictive value (PPV) and negative predictive value (NPV) of the respective cut-off points were calculated. Odds ratios (OR) from the univariate analyses were calculated for each cut-off point. A two-tailed *p* value < 0.05 was considered statistically significant. Ninety-five percent confidence intervals (95%CI) were calculated when applicable. Statistical analysis was performed using SPSS version 25 (IBM Corp., New York, NY, USA).

### 2.5. Laboratory Methodology

POsm and UOsm were measured by A2O^®^ osmometer (Advanced Instruments, INC, Norwood, MA, USA) by freezing-point. AU5800^®^ Analyzer (Beckman Coulter, Indianapolis, IN, USA) was used to measure serum and urinary electrolytes by indirect potentiometry (inter-assay variability coefficient < 0.01). SC was measured by compensated Kinetic Alkaline Picrate Method, AU5800^®^ Analyzer (inter-assay variability coefficient of 0.03 with SC < 0.77 mg/dL and of 0.01 with values > 1.78 mg/dL). 

## 3. Results

A total of 122 patients with hyponatremia were studied: 70 (57.4%) with SIAD, 52 with HH. The median age: 79 years (70–85), 57 (46.7%) were women. Median SNa of the hyponatremic episode: 130 mmol/L (126–133). In all patients, serum glycemia was consistently below 140 mg/dl, and correction of SNa for glycemia was not required. 

Nine of the 70 SIAD patients (12.8%) also presented clinical data of polydipsia (fluid intake ≥ 3 L/day in the absence of thirst), presenting a UOsm > 100 mOsm/L but lower than POsm. Urinary losses were present in 50/52 HH patients (96.2%), gastrointestinal losses in 5/52 patients (9.6%), and hemorrhage in 4/52 patients (7.7%). The exact cause of HH remained unknown in 2/52 patients (3.8%). The five patients with gastrointestinal losses and the four patients with hemorrhage also presented concomitant urinary losses. Of the 50 patients with urinary sodium loss, 15 were related to diuretic use, 5/50 to Addison’s Disease, 38/50 to isolated acquired hypoaldosteronism. Serum aldosterone (PA) and direct renin (DR) values were available in 33/38 patients with hypoaldosteronism. The median PA value was 79 pg/mL (IQR: 33 to 176), and median DR was 23 pg/mL (IQR: 12 to 51), with normal values for PA in normokalemia ranging from 90 to 200 pg/mL and normal DR values from 8 to 20 pg/mL at recumbent position. Patients were classified as presenting an aldosterone deficit when levels were low in spite of hyperkalemia, and as presenting aldosterone resistance when levels were elevated. Table 2 shows patients’ parameters in eunatremia and hyponatremia classified by volemic status when hyponatremic. Parameters coinciding with eunatremia up to 3 months before diagnosis of hyponatremia were available in 42/52 (80.8%) hypovolemic patients and in 52/70 (74.3%) euvolemic patients.

**Figure 1:**
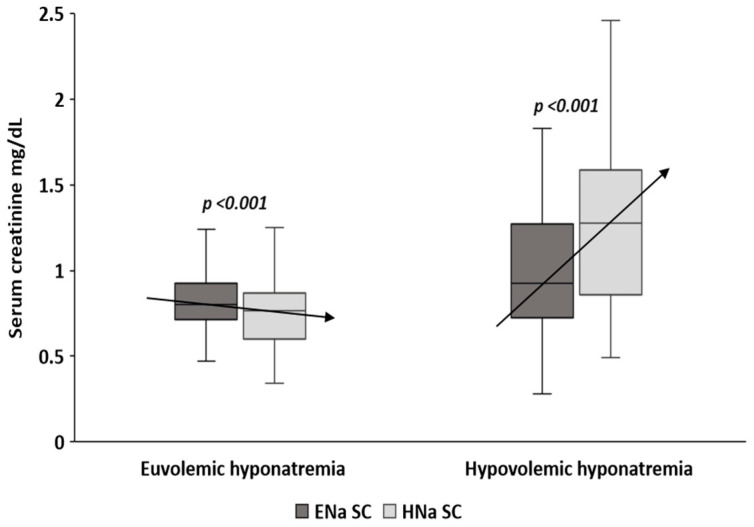
∆SC from eunatremia to hyponatremia according to volemic status during hyponatremia. The arrows indicate the trend of the change in SC during the hyponatremic episode when compared with eunatremia. ENa SC: serum creatinine coinciding with eunatremia. HNa SC: serum creatinine coinciding with hyponatremia.

**Figure 2:**
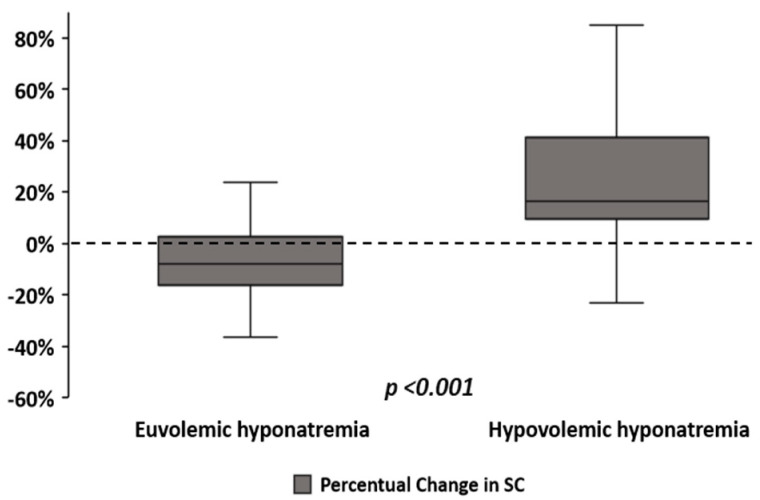
Median of ∆SC% from eunatremia to hyponatremia, classified according to volemic status during hyponatremia. SC: serum creatinine.

The median SNa fall during hyponatremia was 8 mmol/L (5–11) in EH, and 9 mmol/L (4–14) in HH (*p* = 0.317). SC elevation during HH was more marked than the descent observed during EH (Figure 1). Both median ∆SC and ∆SC% were negative in EH but positive in HH (Figure 2), this difference was statistically significative (Table 2). We found a correlation between ∆SC and the SNa decrement in subjects with HH (r: +0.493, *p* < 0.001), but not in the EH (r: −0.082, *p* = 0.501). 

Both HH and EH groups had cases with a positive ∆SC, but at a higher rate in HH than in EH patients (90.4% vs. 25.7%, *p* < 0.001). Furthermore, in patients with a positive ∆SC, the median of ∆SC was higher in HH than in EH (0.19 vs. 0.07 mg/dL, *p* < 0.001), as was the median of ∆SC% (22% in HH vs. 8% in EH, *p* = 0.001).

The rate of cases showing a decrease/null change in SC during the hyponatremia was higher in the EH than in the HH (74.3% vs. 9.6%, *p* < 0.001). In patients with a decrease/null change in SC, median ∆SC was lower in EH than in HH (−0.12 vs. −0.02 mg/dL), although this difference was not significant (*p* = 0.083). However, median ∆SC% was significantly lower in EH than in HH (−12% vs. −2%, *p* = 0.033). 

**Figure 3:**
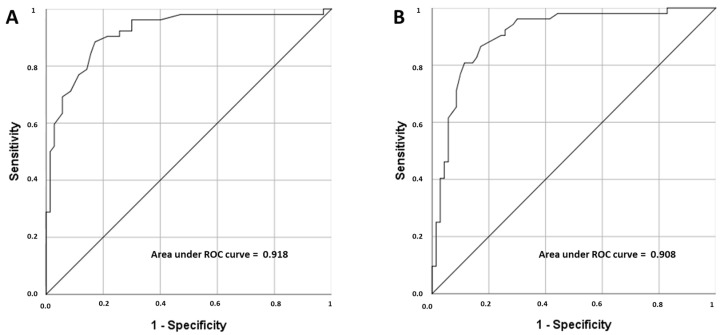
Area under ROC curves for ∆SC (**A**) and ∆SC% (**B**), evaluating their accuracy for distinguishing between hypovolemic hyponatremia and euvolemic hyponatremia. ROC: receiver operator characteristic, ∆SC: change in the SC from eunatremia to hyponatremia, ∆SC%: percentual change in the SC from eunatremia to hyponatremia.

ROC curves for ∆SC and ∆SC% are displayed in Figure 3. In the entire group, ROC curve analysis found an AUC of 0.918 (95%CI: 0.865 to 0.970, *p* < 0.001) for ∆SC, and an 0.908 (95%CI: 0.853 to 0.962, *p* < 0.001) for ∆SC%, to differentiate HH from EH. The calculated OR, sensitivity, specificity, PPV and NPV of the optimal cut-off points of SC and ∆SC% to classify patients as hypovolemic or euvolemic, are shown in the Table 3.

UNa was measured in 48/52 HH patients (92.3%) and in 63/70 EH patients (90%) coinciding with initial volemic assessment. If we had used the cut-off point of UNa ≤ 30 mmol/L to classify the patients as hypovolemic, only 16.7% of the HH patients would have been identified. Furthermore, 7.9% of the EH patients would have been mistakenly classified as hypovolemic. ROC curve analysis for UNa as tool to identify HH found an AUC of 0.544 (95%CI: 0.434 to 0.653, *p* = 0.432) for the cut-off point ≤ 30 mmol/L. The sensitivity, specificity, PPV and NPV of this cut-off point were as follows: 16.7%, 92.1%, 61.5% and 59.2%, respectively.

## 4. Discussion

Our results indicate that even the detection of small changes in SC—although SC remains within normal limits—can be useful for the determination of volemic status in patients with hyponatremia, specifically to distinguish hypovolemia from euvolemia. In fact, a ∆SC ≥ 0.11 mg/dL or a ∆SC% ≥ 10% indicate HH with a probability of 90% or 86.4%, respectively. A ∆SC ≤ −0.05 mg/dL or a ∆SC% ≤ −3% on the other hand, indicate EH, with a probability of 95.5% or 96.1%, respectively. These findings are coherent with a biological plausibility. In HH, intravascular water and ECV are reduced [2,4,14], whereas in EH, intravascular water and ECV can be normal or elevated [2,4,18,19]. Thus, in HH, SC can rise as SNa descends, whereas in EH, both SC and SNa can descend together, or SC can remain unchanged.

Clinical guidelines recommend assessment of volemic status as a first step in the diagnosis of hyponatremia [1,2]. Although measurement of the HIJP by physical inspection could be definitive for volemic classification [15,20], and has been validated by ultrasound studies [21,22], not all clinicians have experience in assessing normal or low levels. In fact, many studies have found difficulties and lack of precision in the clinical assessment of volemic status [3,5,9]. Thus, alternative methods have been proposed to facilitate volemic classification. However, overlaps and/or the need for additional urine studies for their application can limit their clinical usefulness. 

A fractional excretion of uric acid (FEUA) > 12% has shown an adequate accuracy for distinguishing EH caused by SIAD from non-SIAD hyponatremia in patients treated with diuretics [10]. However, the results displayed important overlap between euvolemic and hypovolemic subjects. In fact, a high FEU is also observed in other causes of HH, such as cerebral salt-wasting [23]. Other markers, such as the fractional excretion of urea, have also shown marked overlap when comparing hypovolemic to euvolemic patients [10].

Our results indicate that measurement of UNa, the most frequently used biochemical tool for volemic assessment in hyponatremia, has serious limitations, particularly when hypovolemia is induced by renal losses of sodium as in the most of our cohort. A high percentage (83.3%) of subjects with HH presented elevated UNa, which would have been suggestive of euvolemia. In fact, we found a PPV of only 61.5% for a UNa ≤ 30 mmol/L in diagnosis of HH. The accuracy of UNa in differentiating EH from HH has been widely studied, and the cut-off point of >30 mmol/L proposed to be an acceptable indicator of EH. In prior studies, sensitivity estimates ranged from 87 to 100%, but specificity estimates ranged from 52 to 83% [5,9,10,11], resulting in hypovolemic versus euvolemic patients overlap, as we found. The explanation is that high UNa levels can be detected not only in SIAD, but also in HH with urinary sodium loss, as occurs with diuretic use, bicarbonate administration, Addison’s Disease, isolated acquired hypoaldosteronism, or cerebral/renal salt wasting. In our series, 50 patients had at least one clinical factor for urinary sodium loss. Of them, 38 had isolated acquired hypoaldosteronism. We have previously reported in the 18th European Congress of Endocrinology that isolated acquired hypoaldosteronism can induce HH [24]. The 38 patients with acquired hypoaldosteronism of the current study were included in that report. Based on our data, drug-induced hypoaldosteronism would be the main cause [24]. One of the drugs most related with hypoaldosteronism is trimethoprim [25,26,27,28]. Additionally, trimethoprim increases the SC by inhibiting tubular secretion of creatinine. Based on this, one can suppose that our findings could be biased if several of the patients in the current study were treated with trimethoprim. However, only 9/52 hypovolemic and 2/70 SIADH patients in our series were under trimethoprim (data not shown). Therefore, our analysis was not significatively affected by the presence of a treatment with trimethoprim. It is to resalt that isolated hypoaldosteronism as a cause of hyponatremia has rarely been reported. Given this fact, our experience could be different from that of other specialty referral clinics.

The same conditions that interfere with the UNa rentability also limit the usefulness of the fractional excretion of sodium [11]. Furthermore, euvolemic states such as primary polydipsia, or SIAD—when accompanied by a low sodium intake—can present low Una [29]. Therefore, a UNa ≤ 30 mmol/L does not always adequately distinguish patients with HH from those with EH in some clinical situations.

Although another biochemical method, the serum urea nitrogen-to-creatinine ratio, used to be applied to determine a hypovolemic status, the usefulness of this parameter has not been validated. A prior study [30] did not find an adequate accuracy of this parameter to distinguish between prerenal from intrinsic acute kidney injury, the former caused by hypovolemia, with an AUC of 0.5. Therefore, the use of this method in the setting of volemic assessment would not be adequate.

The response of SNa to isotonic saline solution (ISS) infusion has often been considered as a gold standard, considering hypovolemia when a SNa ascends > 5 mmol/L after 2 L ISS infusion over 24 h [9]. However, the response of euvolemic and hypovolemic subjects overlaps [11,13]. EH patients can show marked ascent of SNa following ISS infusion [13]. Furthermore, since EH patients can also display a frank SNa descent [13], this test can be dangerous, as the response to ISS cannot be uniform in HH. In patients weighing ≤ 87 kg, an ISS infusion of 2 L (≥23 mL/kg/day) would induce an SNa increase > 5 mmol/L. However, in subjects with a higher weight, the probability of achieving this SNa ascent is lower [31].

Impedance measurement has also been proposed for volemic assessment in hyponatremia [32]. Although Cumming et al. suggested its superiority to clinical examination in elderly patients [33], several limitations regarding the volemic evaluation have been encountered in their study. In contrast, our group found that physical inspection of the maximum HIJP is a clearly better tool for clinical decision-making than impedance [34]. These last findings are in accordance with the study by Vasko et al. [35], where clinical judgment was found as the best element for overhydration assessment. Additionally, an impedance device is not widely available in all hospital settings. 

Measurement of SC, however, is readily available, and easily interpreted by clinicians. Furthermore, many patients will have accessible prior SC levels for comparison with those of the current hyponatremic episode. When previous SC levels are not available, the short-term variation of SC together with that of SNa following therapy can be used. We must point out that hypervolemic hyponatremia must be previously ruled out, since SC changes can be similar to those observed in HH. 

The main limitations of our study are its retrospective design, and the lack of a specific evaluation of patients’ nutritional status, a factor that can influence SC. We tried to minimize the latter through the selection of a short time interval for comparing SC. Thus, in these outpatient subjects, there would be a low probability of a marked change in body muscle. Anther limitation in the retrospective design is the fact that we could not evaluate the effect of comorbidities and other therapies administered. Furthermore, it is important to note that the sensitivity and specificity of UNa and ∆SC found in our study may differ in patients encountered in general outpatient practices or on hospital wards, where the frequency of the urinary causes of hypovolemia could be below that of our study. The fact that our cohort contains an important number of cases of isolated hypoaldosteronism, a disease rarely reported as a cause of hyponatremia by other authors, could contribute to differences between our data and those other specialty referral clinics. Additionally, if the clinical evaluation performed by physicians during their medical practice was inaccurate, our analyses of sensitivity and specificity would not be valid. However, the accuracy of the evaluation of volemia using the maximum HIJP has been validated [21,22,36,37], and this method used in a majority of our patients. Another limitation is that a power analysis for sample size calculation was not done.

An important strength of this study is the robust volemic classification of patients. In addition to the parameters used for the determination of volemic status, volemia was confirmed by assessment of clinical response to therapy.

## 5. Conclusions

We have shown that the assessment of changes in SC from eunatremia to the hyponatremic episode is useful in distinguishing between HH and EH. Small percentual changes in SC, of the order of ≥10% or ≤−3%, present a high accuracy for the classification of hyponatremic patients as hypovolemic or euvolemic, respectively. Furthermore, since the determination of SC is widely available, its assessment can be used in a wide range of clinical settings.

## Figures and Tables

**Table 1:** Clinical parameters used to define hypovolemia.

	Hypovolemic Patients (N = 52)
Thirst, n	38
Orthostatism, n	21
Decreased eye tone, n	48
Hypotension, n	2
Tachycardia, n	6
HIJP below the sternal angle, n	38
UNa, ≤30 mmol/L, n	8
Criterion 1 for hipovolemia, n *	38
Criterion 2 for hypovolemia, n **	14

HIJP: height of the internal jugular pulse. UNa: urinary sodium. * Criterion 1: presence of a HIJP below the sternal angle along with at least two of the following: thirst, orthostatic symptoms, hypotension (blood pressure ≤ 90/60 mmHg), tachycardia (heart rate ≥ 90 bpm), decreased eye tone on palpation, or spot UNa ≤ 30 mmol/L. ** Criterion 2: when HIJP was not evaluated, the presence of at least three of the other clinical features described above defined hypovolemia.

**Table 2:** Patients’ parameters in eunatremia and hyponatremia as classified by volemic status when hyponatremic.

	Euvolemic Hyponatremia (N = 70)	Hypovolemic Hyponatremia (N = 52)	*p* ^a^
Age, years	80 (72–86)	77 (67–84)	*0.15*
Female, n (%)	38 (54.3)	19 (36.5)	*0.05* *
**Eunatremia**			
SNa, mmol/L	138 (137–140)	138 (137–139)	*0.44*
SC mg/dL	0.8 (0.71–0.94)	0.93 (0.72–1.34)	*0.01* *
**Hyponatremic episode**			
SNa, mmol/L	131 (128–133)	129 (124–133)	*0.23*
SK, mmol/L	4.5 (4.3–4.8)	5.1 (4.6–5.3)	*<0.001* *
POsm, mOsm/kg	272 (267–282)	280 (275–288)	*0.002* *
SC, mg/dL	0.76 (0.6–0.87)	1.28 (0.83–1.64)	*<0.001* *
*p* ^b^	<0.001 *	<0.001 *	
∆SC, mg/dL	−0.07 (−0.15–0.02)	+0.18 (0.09–0.39)	*<0.001* *
∆SC%	−8 (−16.2–2.5)	+16.5 (10–41.3)	*<0.001* *
UNa, mmol/L	91 (61–124)	58 (38–79)	*<0.001* *
UK, mmol/L	32 (23–52)	28 (23–41)	*0.51*
UOsm, mOsm/kg	433 (328–581)	375 (308–470)	*0.03* *

Quantitative variables are expressed in medians and [interquartile range]. SNa: serum sodium, SK: serum potassium, POsm: plasma osmolality, SC: serum creatinine, ∆SC: change in the SC from eunatremia to hyponatremia, UNa: urinary sodium, UK: urinary potassium, UOsm: urinary osmolality. ^a^ Euvolemic hyponatremia vs. Hypovolemic hyponatremia. ^b^ Hyponatremia vs. eunatremia. * *p* < 0.05.

**Table 3:** Odds ratio, Sensitivity, Specificity, Positive predictive and negative predictive values of optimal cut-off points of ∆SC and to classify patients as hypovolemic or euvolemic.

	SS (%)	SP (%)	PPV (%)	NPV (%)	OR * [95%CI]	*p* *
**∆SC Cut-off point**						
For hypovolemic hyponatremia:						
≥0.11 mg/dL	69.2	94.3	90	80.5	37.1 [11.5 to 119.4]	*<0.001*
For euvolemic hyponatremia:						
≤−0.05 mg/dL	60	96.2	95.5	64.1	37.5 [8.4 to 166.7]	*<0.001*
**∆SC% Cut-off point**						
For hypovolemic hyponatremia:						
≥10%	73.1	91.4	86.4	82.1	29.0 [10.3 to 81.7]	*<0.001*
For euvolemic hyponatremia:						
≤−3%	70	96.2	96.1	70.4	68.3 [13.0 to 262.2]	*<0.001*

CI: confidence interval, ∆SC: change in SC from eunatremia to hyponatremia, SS: sensitivity, SP: specificity, PPV: positive predictive value, NPV: negative predictive value, * Odds Ratio obtained from the univariate logistic regression analysis.

## Data Availability

Data available on request due to restrictions e.g., privacy or ethical.
